# Teaching Comprehensive Geriatric Assessment (CGA) in medical education: a scoping review

**DOI:** 10.1007/s41999-025-01157-4

**Published:** 2025-03-07

**Authors:** Regina Roller-Wirnsberger, Carolin Herzog, Sonja Lindner-Rabl, Mathias Schlögl, Maddalena Illario, Maria Cristina Polidori, Katrin Singler

**Affiliations:** 1 Department of Internal Medicine, Research Unit Services for Old Age and Lifelong Health, Medical University of Graz, Graz, Austria; 2 Division of Geriatric Medicine, Clinic Barmelweid, Barmelweid, Switzerland; 3 Department of Public Health, Federico II University, Naples, Italy; 4 Ageing Clinical Research, Department II of Internal Medicine and Center for Molecular Medicine Cologne, Faculty of Medicine and University Hospital Cologne, Cologne, Germany; 5 Cologne Cluster of Excellence on Aging and Aging-associated Diseases (CECAD), University of Cologne, Cologne, Germany; 6 Institute for Biomedicine of Ageing, Friedrich-Alexander University Erlangen-Nürnberg, Nuremberg, Germany; 7 Department of Geriatrics, Klinikum Fürth, Fürth, Germany

**Keywords:** Comprehensive Geriatric Assessment, Education and training, Medical doctors, Older adults, Competence development

## Abstract

**Purpose:**

To gather and summarize evidence on educational and training formats for medical doctors in performing Comprehensive Geriatric Assessment (CGA) in under- and postgraduate medical education.

**Methods:**

A comprehensive literature review was conducted using the databases Medline, CINAHL, Cochrane and Embase to identify educational intervention studies and cohort studies related to CGA education and training for medical professionals. Additional references were incorporated through reference tracking. Studies included were then grouped according to competence level addressed during CGA trainings to create a current competence-based framework on educational tools to teach CGA to medical students and doctors.

**Results:**

Sixty eligible studies were identified, with 42 addressing the full implementation of CGA and 18 focusing on specific CGA domains. High variability in duration, intervention design and evaluation methods was consistently observed across the included studies.

**Conclusion:**

The findings underscore the need for further coordinated research in CGA education and training to consolidate evidence and pave the way to more innovative, high-quality healthcare systems capable of addressing the complexities of an aging society.

**Supplementary Information:**

The online version contains supplementary material available at 10.1007/s41999-025-01157-4.

## Key summary points

**Aim:**

To provide evidence on effectiveness on education and training formats for Comprehensive Geriatric Assessment (CGA).

**Findings:**

Sixty studies were included, showcasing highly heterogeneous education and training formats offered to under- and postgraduate learners of CGA performance.

**Message:**

To accelerate and optimize the implementation of CGA in clinical practice, further studies are required using more homogeneous methods, larger samples size and adequate endpoints.

**Supplementary Information:**

The online version contains supplementary material available at 10.1007/s41999-025-01157-4.

## Background

Comprehensive Geriatric Assessment (CGA) is a multidimensional, interdisciplinary diagnostic process designed to evaluate the medical, psychologic, and functional capabilities of older adults. The primary objective of CGA is to enable the development of a coordinated and integrated treatment plan, along with long-term follow-up across various care settings [1]. CGA is the technological cornerstone of geriatric medicine, grounded in a specialised interdisciplinary team approach, including medical doctors (MDs), nurses, social workers, physical and occupational therapists, dieticians, pharmacists, psychologists, to name some of the professions involved [2]. To standardize this complex assessment process, validated tools e.g. mobility assessments, nutritional and psychologic evaluation etc., have been developed and incorporated into a standardized assessment tool battery [3]. Based on CGA, several tools have been developed in the last decades, ranging from multidomain but basic screening qualitative tools to highly validated, scaled instruments enabling inter-professional co-management, clinical decision-making and integrated care [4]. CGA constitutes the very core of geriatric competence and its structured use and purpose are object of two most recent national guidelines [5, 6].

In clinical settings, medical doctors develop care plans for the management of older people and lead interprofessional teamwork, while enabling, in a flat-hierarchical, genuine interdisciplinary co-management, the exchange needed to carry out a tailored diagnostic and therapeutic care plan. Geriatricians are also responsible for communicating with patients, their families and caregivers, diagnosis and therapeutic options to offer an individualized plan in shared decision-making [7]. Needless to say, such a multifaceted set of tasks demands a broad portfolio of competencies necessary for inter-professional collaborative practice (ICP) [8].

Consequently, MDs should be equipped not only with the clinical skills required to perform CGA but also with the communication and management capabilities essential for this role. The development of such professional capabilities [9] necessitates the integration of geriatric training throughout both undergraduate and postgraduate medical education. While training recommendations, including learning objectives and entrustable professional activities (EPAs), are available for medical students and residents in geriatric medicine [10, 11], there remains a lack of clarity regarding the most effective training formats to achieve the proposed learning goals at various levels of competency and professionalism in performing CGA within an ICP setting. This gap in knowledge contributes to significant variability in the timing and structure of CGA training across medical curricula worldwide, and therefore of CGA adoption and implementation on the large needed scale.

To close this knowledge gap, the present review was designed to comprehensively address scientific evidence on CGA training formats and scenarios offered to under- and postgraduate learners of CGA performance.

## Methods

Given the anticipated volume of literature on this broad topic, the authors elected to conduct a scoping review to comprehensively map the existing research.

As part of this, a comprehensive literature review was performed focusing on educational intervention studies and cohort studies related to the training of CGA for medical professionals, published between January 2000 and April 2024. Articles in English were searched across several databases, including Medline (via PubMed), CINAHL (via EBSCOhost), Cochrane Libraries (via Ovid), and Embase (via Ovid). Our search strategy incorporated keywords and controlled vocabulary terms, such as the MeSH headings “professional education” OR “education and training” AND “comprehensive geriatric assessment” OR “geriatric assessment” (see search protocol as supplementary information). Where necessary, controlled vocabulary terms were adapted to fit the specific database options by utilizing relevant synonyms. In addition, a search for grey literature was performed using Google Scholar. Further studies were identified through reference tracking of the included articles. Two researchers independently conducted the title/abstract screening followed by a full-text review. In cases of disagreement, a third reviewer was consulted to reach a consensus.

Studies were included if they met the following criteria: 1) randomized controlled trials, cohort studies or quasi-experimental educational intervention studies; 2) focused on undergraduate or postgraduate medical professionals, including medical students, interns, residents, geriatric fellows or other physicians; and 3) involved education and/or training in CGA as the intervention. The primary outcome of interest was the effectiveness of the educational interventions, as measured by improvements in knowledge, attitudes, clinical skills/performances, competencies, and/or patient-related outcomes. Studies that assessed outcomes through both trainee self-assessment and/or evaluations by educational providers were included.

Data charting was performed by one author and checked by a second author. The extracted data was presented in tabular form using MS Office Word software.

In a next step, authors grouped the training formats used in the publications included into different competence levels to facilitate a comprehensive overview and support choices for readers during preparing their educational interventions. To do so, authors made use of the concept of competence development, also known as the “*conscious competence model”* [9]. This model provides a framework for understanding the learning process in medical education. It comprises of four stages: The beginning of a learning cycle is characterized by “unconscious incompetence”. At this stage, learners are unaware of their knowledge and/or skills gaps. They are not aware of the competence gaps, which limit learners’ intrinsic motivation to learn. As learners become more aware of their deficiencies, they realize their need for support and further education. This stage is called “conscious incompetence”. At the stage of “conscious competence”, knowledge and skills, however, start to focus on application of these in practice. This implies that competences are present, not yet fully automated and applicable in an entrusted way. In the final phase of this model (“unconscious competence”), learners can perform tasks effortlessly without need to consciously think about them. Authors grouped training formats around this model, not taking in consideration assessments described in the publications to align CGA training opportunities along a learning journey of different levels of competences.

## Results

The search strategy identified a total of 1,122 studies. After removing duplicates (*n* = 85) and excluding studies based on title/abstract (*n* = 838) and full-text screening (*n* = 139), 60 studies were included. These consist of 42 investigations on the full CGA, holistically including the different assessment domains [12–53] and 18 studies on specific CGA domains, in which only certain, predominantly functional and/or cognitive capabilities of older adults were evaluated [54–71].

**Fig. 1:**
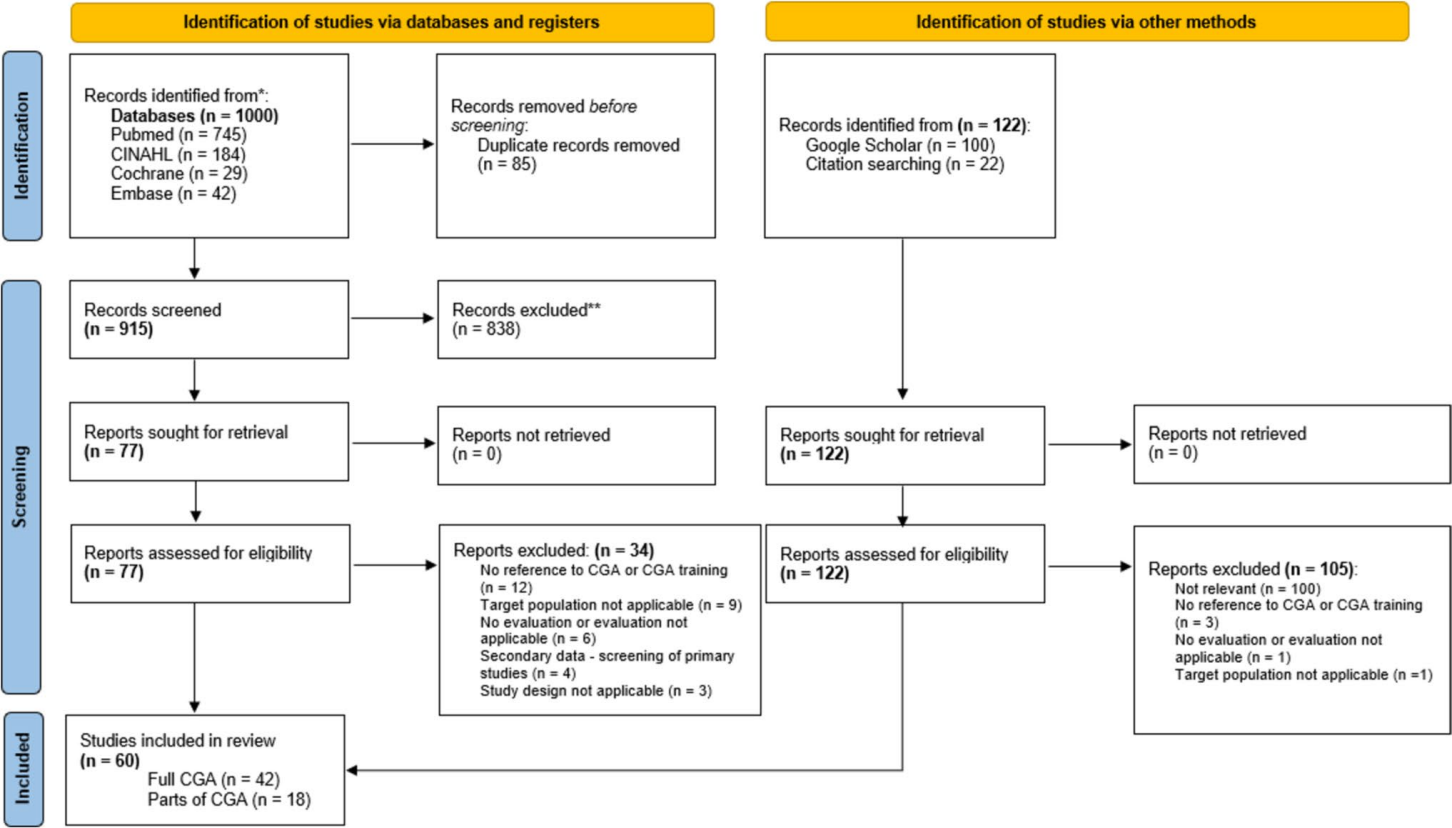
Studies screening algorithm. A total of 60 studies, composed of 42 studies considering full CGA and 18 studies covering particular domains of a CGA, were included in the present review

The PRISMA 2020 flow diagram [72], as shown below in Fig. 1, illustrates the screening process.

Of the 42 studies on the full CGA, 26 focused on training of medical students at various levels of their undergraduate training [13–38], 13 of residents and interns [12, 39–50], 1 on both groups [51], and 2 on continuous professional development programs [52, 53]. For training on specific CGA components, 15 studies were identified for undergraduate programs [54–68] and 3 for resident training [69–71]. Details of these educational studies, including those covering the full CGA and those focused on specific components, can be found in Tables 1 and 2 in the supplementary material.

In total, 6,449 students, ranging from first-year to fifth-year trainees, were included. However, three studies on undergraduate students [36, 59, 67] did not report participant numbers. The number of residents included in CGA training studies was significantly lower, totaling 1,276, with two studies failing to specify the number of participants [45, 53]. The duration of training interventions varied considerably across the included studies, comprising different time formats. Courses and/or programs incorporating geriatric content ranged in general from brief sessions of approximately 30 min [64] to comprehensive programs lasting up to four years [17]. The reported length of training formats can be categorized more specifically as follows: minutes (approx. 30 min) [64], hours (range: 1–64 h) [15, 16, 18, 25, 42–44, 54, 55, 57, 59, 62, 71], days (range: 0.5–3 days) [13, 29, 32, 39–41, 52], weeks (range: 1–10 weeks) [14, 19–23, 26, 34, 35, 37, 58, 66], months (range: 1–12 months) [30, 46, 48–51, 70] and years (range: 1–4 years) [17, 45, 60]. In this context, it is worth mentioning that a great number of training programs last 4 to 4.5 weeks [20, 21, 35, 66] or equivalent 1 month [30, 46, 49–51] and are mostly conducted as (part of) a clerkship/rotation. However, not all studies provided precise information regarding the total time required for the training. In particular, the presence of studies from different countries and continents (Europe *n* = 5, North and South America *n* = 51, Australia *n* = 2, Asia *n* = 1, Transnational *n* = 1) underlines the importance of the topic across geographic borders, with North America being by far the frontrunner in terms of published included studies (*n* = 49). In this context, however, country-specific factors must also be taken into account, whereby divergent (medical) education systems can make it difficult to align the underlying focus based on the studies included (status quo of teaching geriatrics, geriatric priorities).

There were no differences in the training formats between studies that addressed the full CGA and those that focused on individual CGA components. None of the studies provided detailed learning objectives for CGA performance. Instead, they mainly outlined the competence level expected after training. This heterogeneity makes it difficult to summarize a robust evidence-base for educational formats for different level of competences during training to be used efficiently. What may be seen from the training formats tested and enclosed into this review is a consistently high competence level addressed within most of the studies. However, the assessment tools chosen to prove efficiency of didactic formats tested in the studies did not address the competence level during training [73]. In other words, assessments evaluated knowledge gain only while addressing higher performance levels during the training sessions.

It is also interesting to see, that none of the studies included explicitly targeted team working capacities and/or collaborative practice skills as mandatory for the intrinsic mission of performing CGA and explained earlier in this paper [8]. At least this has not been outlined explicitly in the description of the different programs, nor has it been tested through the assessment instruments included in the studies. Given the nature of CGA performance in an inter- or multi-professional team together with other care providers, these findings are interesting.

**Fig. 2:**
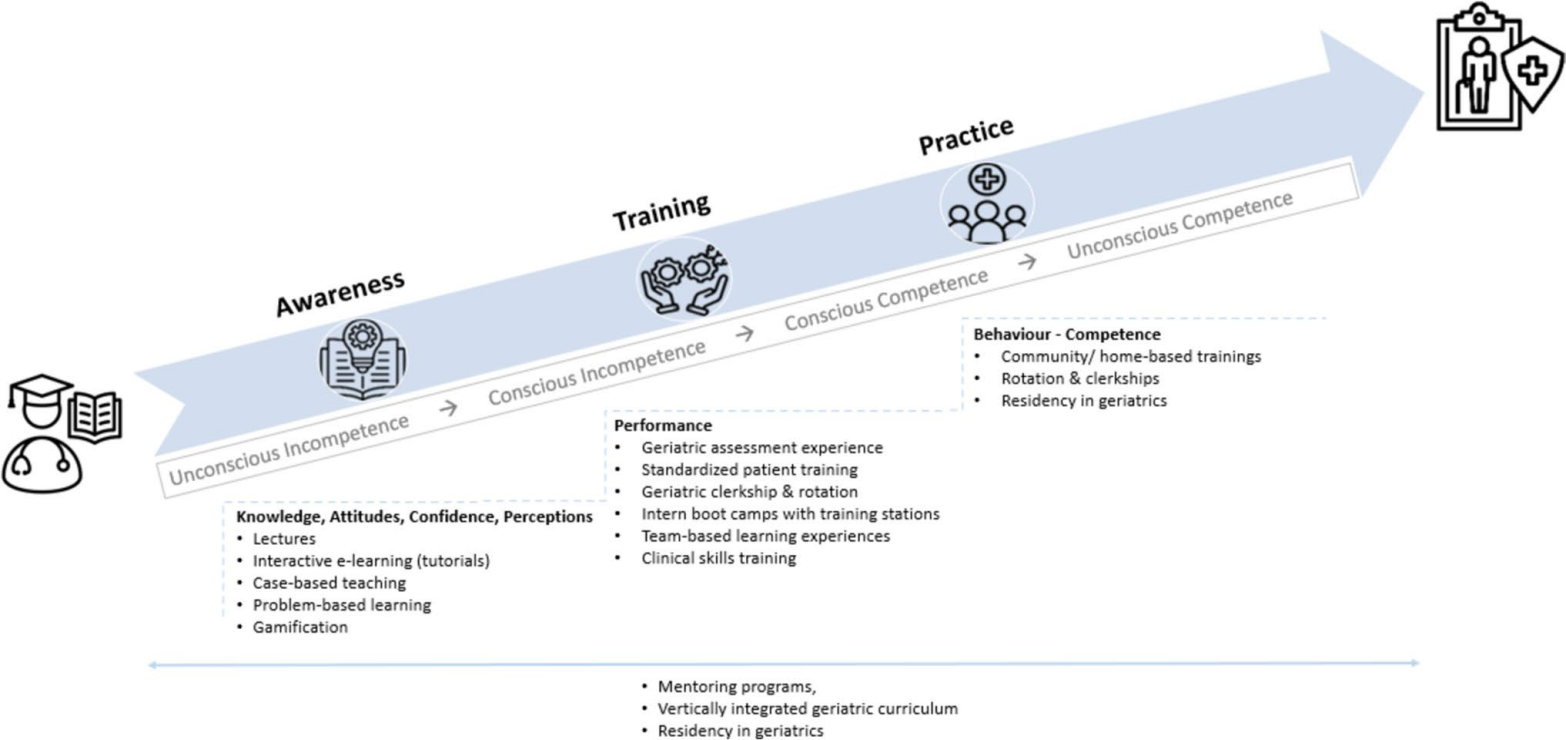
Illustrates a learning path inspired by a competence-development framework by Keeley and colleges [9] to master skills of performing CGA

Grouping results around the *conscious competence model* published by Keeley and colleges [9] authors could showcase, that multistep educational approaches seem to align with the aims and goals for training of CGA in a most appropriate way. Figure 2 aims at outlining this observation. During the “novice period in medical education” it seems important to create awareness for the topic of evidence-based care of older patients with complex care needs. Lectures [16, 59, 70], e-learning offers [31, 64, 68], case based teaching [31, 39, 42, 57, 59, 68, 70] and problem based learning [18, 19] seem to efficiently address leaners’ needs in this period of medical education. To translate this basic knowledge and skills into practice standardized training opportunities such as clinical skills training [18], rotations [22, 26, 39, 43, 46–51, 58], boot camps [55] and team-based learning experiences [59] have been described in literature. To support unconscious competence during daily clinical practice and performance of teamwork based on CGA, however, rotations in homecare settings [38, 61], rotations, residencies and clerkships in clinical settings with geriatric focus [22, 30, 35, 39, 40, 43, 44, 46, 50, 51, 58, 66, 70, 71, 74] seem necessary. There seems to be evidence out there, that mentoring programs, vertically integrated curricula and mandatory residencies and clerkships in geriatric environments have a strong and sustainable educational impact on the competence or working with CGA in clinical practice [17, 33, 60, 63, 66].

The figure above shows the current evidence on didactic approaches tested in CGA training and outlined in the studies included in this review.

## Discussion

Authors have recently published the current evidence on inter-professional education for disciplines other than MDs involved in conducting CGA [2]. In contrast, the present publication specifically focuses on the education and training of MDs in performing CGA, aiming to support academic geriatricians actively involved in education and training of MDs in this specialized field. The review assessed various training formats for students and residents at different competence levels, aiming to raise awareness of all MDs and students for the use of CGA in clinical practice. Beyond this scope, authors also tried to create an evidence base on efficacy of training formats for CGA at advanced levels of trainings for those doctors, who need to be able to perform, interpret, and discuss CGA effectively.

This scoping review could demonstrate that there are different training opportunities described for different competence levels of trainees. Formats mainly used during undergraduate medical trainings across Europe, such as lectures [16, 59, 70], e-learning offers [31, 64, 68], case-based teaching [31, 39, 42, 57, 59, 68, 70] and problem-based learnings [18, 19] are supporting awareness building and knowledge retention. Reflecting this evidence, however, towards the curriculum in geriatric medicine recommended by the European Geriatric Medicine Society (EuGMS) [10], this approach will most likely not be able to prepare our medical students efficiently for the competence levels to understand and reflect results from CGA as recommended by geriatric experts. This is an important finding, as aging populations are observed in low- to middle and high-income countries globally. CGA is a key quality element in the person-centered care model for older people and every profession, especially MDs, should be capable of interpreting CGA results, discussing them with colleges and designing a corporate care plan based on patients’ goals and capacities [1].

Beyond professional standards and ethics, effective patient-physician relationship, inter-professional teamwork, role redefinition, and communication are crucial for integrated management and organization in CGA. While CGA is often used as a mechanistic scoring instrument in many healthcare systems, its role as a key aspect of geriatric medicine extends beyond mere scaling of functions. CGA should be viewed as central to inter-professional collaboration and person-centered care, regardless of the care setting. Understanding how best to equip current and future MDs with the necessary core competencies is essential, although targeted educational approaches to CGA remain challenging. Education and trainings need to bridge the gap between standardized assessments on the one hand and individualized and rather flexible methods, also including goal-oriented care planning on the other hand.

In this context, the importance of competency-based education has been thoroughly discussed in the literature. In a recently published model [75], the interplay of three layers that build a framework for competence conceptualization was discussed. Medical competence ideally consists of knowledge and skills that are required independent from professions; knowledge, skills and attitudes that are acquired within health care practices and, finally, competences that are integrated into an individual’s personality, which cannot be standardized and contribute to a dynamic and diverse health care workforce. In addition, competency-based education allows priority setting in health care and offers a mechanism to incorporate contextual health needs with health workforce development.

Our recent work already demonstrated the effects of advanced inter-professional training methods for professions involved in CGA and not being MDs, to maximize the impact of CGA in clinical practice [2]. However, beyond inter-professional training offers, geriatricians should consider their role in shaping future generations of MDs. The most important finding of our work in the context of medical education is the effectiveness of vertically integrated training programs for CGA, utilizing diverse didactic tools (see Fig. 2) [63]. Mentoring programs, rotations, and clerkships with geriatric focus appear to enhance training outcomes [17, 22–24, 26, 30, 31, 33, 35, 38, 39, 43, 46–51, 58, 60, 66, 76]. Virtual teaching and (online) learning management systems pose an essential factor in this regard, as technology-based teaching and learning environments foster education and democratize knowledge in a flexible manner [77].

In a bibliometric review [77], six thematic clusters were identified that highlight important aspects to gain a holistic picture of sustainable virtual teaching modalities: In detail, clusters relate to (1) the prevalent function of technological tools to create virtual teaching experiences, (2) the significance of qualified educators, (3) the necessity of frameworks and governance structures that guide virtual teaching possibilities, (4) a careful consideration of students’ and learners’ experiences and perspectives, (5) the importance of basic technological and digital infrastructure for successful virtual teaching, and finally (6) a needs-oriented integration and adaptation of curricula. There is no doubt that higher education is facing the issue of successfully teaching complex skills to students. Against the previously discussed background, a well-balanced interaction of theory, interventions and technological support promote the achievement of complex thinking and skills, even in particular educational situations and contexts [78], as is often required when addressing specific clinical needs of older adults in different settings.

CGA has been shown to improve outcomes for older adults in both hospital and community settings [79], as it encompasses health and social care needs and promotes multidisciplinary collaboration of various health care professions. A recent paper highlights the need for new care models to integrate CGA for the majority of older patients in non-geriatric hospital units. It also suggests that incorporating geriatric learning objectives into undergraduate training and developing practice guidelines for geriatric syndromes could improve care quality for older patients [80]. Our work may serve as a starting point for connecting geriatricians and interested readers, and for designing research environments to address existing questions.

Despite the extensive number of participants, the studies included in our review has several major limitations. Although a variety of didactic approaches were tested across different competence stages, many training formats were supported by only one or two studies. Another significant limitation is the quality of the included studies. With reference to study and evaluation design, there are mentionable differences in the approach restraining objectivity and comparability of the studies. While a large proportion of studies were only conducted as “uncontrolled” single-arm [12–15, 17–19, 21, 22, 27, 29–35, 40, 41, 43, 45, 46, 48, 49, 51, 52, 54–56, 59, 60, 62, 64–71] or observational research [36, 63], others were carried out as multi-arm studies and/ or used one or more control groups [16, 20, 23–26, 28, 37–39, 42, 44, 47, 50, 53, 57, 58, 61] (e.g., to compare with traditional teaching or using different didactic methods and/or tools) to create better opportunities for comparison. Quality deficiencies are also evident in the evaluation format, as on the one hand a pre-post assessment cannot be found uniformly among all included studies, in some cases the assessment is explicitly carried out merely post-intervention [13, 24, 33, 37, 41, 61, 62, 65, 66, 71], which refers to missing baseline and thus comparing data. On the other hand, there is a high risk of subjective or biased data based on a partially included self-evaluation. In addition to the above mentioned facts, many studies exhibited a mismatch between educational goals and assessment methods [73]. Notably, several studies on residency and internship training, which are primarily workplace-based, focused on lower levels of competence, such as assessing knowledge [40, 44–51, 70, 71] or self-rated confidence in performing CGA [39, 42, 43, 69, 70]. In contrast, undergraduate training studies typically measured knowledge, attitudes, skills [15, 16, 18, 20–22, 25–28, 30, 32–36, 54–56, 59, 61, 63, 64, 66], or professional competence [14, 23, 31], regardless of the students’ year of training. A clear alignment between training formats, participant training levels, and assessment methods was often lacking, posing a significant barrier to interpreting the results. Given the methodologically weak evidence, there is a substantial need for further research in this field.

Based on these insights, authors strongly recommend continued research in the area of geriatric education and training, leveraging the robust networks of geriatricians within Europe and internationally. One potential avenue for progress is through collaboration within the European Geriatric Medicine Society (EuGMS) and the European Union of Medical Specialists– Geriatric Medicine Section (UEMS-GMS), which recently secured a European grant under the COST program, PROGRAMMING CA21122 (https://www.cost.eu/actions/CA21122/). This initiative aims to build networks among stakeholders in older care and geriatrics across Europe and foster implementation of needs-oriented education and training in geriatric medicine.

## Supplementary Information

Below is the link to the electronic supplementary material.

Supplementary file1 (DOCX 109 KB)

Supplementary file2 (PDF 93 KB)

Supplementary file3 (DOCX 46 KB)
