# Supplementary material for: Teaching Comprehensive Geriatric Assessment (CGA) in medical education: a scoping review
Source: Eur Geriatr Med. 2025 Mar 7;16(2):425–33. doi: 10.1007/s41999-025-01157-4 (PMC12014708; doi:10.1007/s41999-025-01157-4)
Supplement: Supplementary file 2 — Supplementary file2 (PDF 93 KB) [file 41999_2025_1157_MOESM2_ESM.pdf]

Review on teaching strategies for performance of Comprehensive Geriatric Assessment (CGA)
European Geriatric Medicine
Roller-Wirnsberger Regina*, Herzog Carolin, Lindner-Rabl Sonja, Schlögl Mathias, Illario Maddalena, Polidori Maria Christina, Singler Katrin
*Medical University of Graz, Department of Internal Medicine, aging-ukim@medunigraz.at

Supplementary Material: Search Protocol

PubMed

04.04.2024

((professional education[MeSH Terms]) OR ("education and training")) AND (((geriatric assessment[MeSH Terms]) OR (geriatric assessment)) OR (comprehensive geriatric assessment))

From 2000 – 2024

745 results

CINAHL

04.04.2024

S1 MH (professional education and training) OR (professional education and training) OR (education and training)

S2 MH geriatric assessment OR geriatric assessment OR MH comprehensive geriatric assessment OR comprehensive geriatric assessment

S3 S1 AND S2

Search Modes: Boolean/Phrase

Apply equivalent subjects

Published Date: 20000101-20240431

184 results

Cochrane via Ovid (Central Register of Controlled Trials and Database of Systematic Reviews)

04.04.2024

1. exp geriatric assessment/

2. "geriatric assessment".mp. [mp=ti, ot, ab, fx, sh, hw, kw, tx, ct]

3. "comprehensive geriatric assessment".mp. [mp=ti, ot, ab, fx, sh, hw, kw, tx, ct]

4. 1 or 2 or 3

5. exp education, professional/

6. "professional education".mp. [mp=ti, ot, ab, fx, sh, hw, kw, tx, ct]

Roller-Wirnsberger Regina*, Herzog Carolin, Lindner-Rabl Sonja, Schlögl Mathias, Illario Maddalena, Polidori Maria Christina, Singler Katrin
*Medical University of Graz, Department of Internal Medicine, aging-ukim@medunigraz.at

7. "education and training".mp. [mp=ti, ot, ab, fx, sh, hw, kw, tx, ct]

8. 5 or 6 or 7

9. 4 and 8

Publication year: 2000 – 2024

29 results

Embase via Ovid

04.04.2024

1. exp geriatric assessment/

2. "geriatric assessment".mp.

3. comprehensive geriatric assessment.mp.

4. 1 or 2 or 3

5. professional education.mp.

6. "education and training".mp.

7. "professional education".mp.

8. 5 or 6 or 7

9. 4 and 8

10. Limit 9 to yr="2000-Current"

42 results

Google Scholar

09.04.2024

"professional education" OR "education and training" AND "comprehensive geriatric assessment"

2000-2024

1470 results

First 10 pages = **100 results**

Review on teaching strategies for performance of Comprehensive Geriatric Assessment (CGA)
European Geriatric Medicine

Roller-Wirnsberger Regina*, Herzog Carolin, Lindner-Rabl Sonja, Schlögl Mathias, Illario Maddalena, Polidori Maria Christina, Singler Katrin
*Medical University of Graz, Department of Internal Medicine, aging-ukim@medunigraz.at
