# Supplementary material for: Teaching Comprehensive Geriatric Assessment (CGA) in medical education: a scoping review
Source: Eur Geriatr Med. 2025 Mar 7;16(2):425–33. doi: 10.1007/s41999-025-01157-4 (PMC12014708; doi:10.1007/s41999-025-01157-4)
Supplement: Supplementary file 3 — Supplementary file3 (DOCX 46 KB) [file 41999_2025_1157_MOESM3_ESM.docx]

**Supplementary Material: Table Study characteristics**

**Table 1** Study characteristics, full CGA (n=42 studies)

| **Author/Year** | **Country** | **Study Design** | **Study Population (and subgroups description)** | **Educational/Training Intervention** | **Primary outcomes of interest** | **Main results** |
| --- | --- | --- | --- | --- | --- | --- |
| ***Undergraduate*** | | | | | | |
| **Goldlist 2022** | USA | Single arm study, post-test | First-year medical and dental students (n=348) | - Educational Geriatrics home visit | - *Confidence and interest in geriatrics* | - Increase in both, interest and comfort/confidence was reported - 70.7% agreed or strongly agreed that the experience increased their interest in the field of geriatrics - 91.7% reported increased comfort assessing functional status; 91.1% for cognitive function; 90.2% for home safety; 88.8% for fall risk and 81.0% for asking about advance directives |
| **Ng 2021** | Malaysia | Single arm study, pre-/post-test | Third-year medical students (n=68) | - Healthcare of Elderly Course | - *Attitude, self-perceived competencies and interest in geriatric medicine* | - Slight, non-significant improvement of attitudes from pre- (3.41±0.36) to post-test (3.45±0.35) (p=0.299) - Significant improvement of self-perceived competencies from pre- (3.62±0.76) to post-test (3.81±0.56) (p=0.009) - No significant difference in interest of geriatric medicine from pre- to post-test (p>0.95) |
| **Lucchetti 2019** | Brazil | Single arm study, pre-/post-test | Third-year medical students (n=166) | - Mandatory course on “Geriatrics and Gerontology”   - In-person practical activities and active theoretical classes | - *Knowledge, attitudes and skills* | - Significant improvement of attitudes (r=0.55-0.81, p<0.001), knowledge (r=0.84, p<0.001), empathy (r=0.51, p<0.001) and clinical skills (r=0.86, p<0.001) |
| **Lucchetti 2018** | Brazil | Controlled, non-randomized study, pre-/post-test | Third-year medical students   - Intervention Traditional Method: n=83 - Intervention Flipped Classroom: n=83 - Control: n=77 | - Mandatory course on “Geriatrics and Gerontology”   - Traditional Lectures (TR) Group   - Flipped-Classroom (FL) Group   - Control Group | - *Attitudes, basic geriatric knowledge and clinical skills* | - Intervention groups improved in all scores (except empathy) compared with control group - Significant better post-intervention improvement of attitude (p=0.049) and knowledge (p=0.035) in FL group compared to TR - No difference between groups in clinical skills |
| **Mendoza De La Garza 2018** | USA | Single arm study (longitudinal), pre-/post-test | First-year – fourth-year medical students   - Knowledge Year 1: n=170 - Knowledge Year 2: n=85 - Attitude Year 1: n=171 - Attitude Year 2: n=91 | - Senior mentor program combined with electronic curriculum | - *Knowledge and attitudes* | - Significant improvement in knowledge from year 1 to year 2 (p<0.001), with no significant differences in attitudes |
| **Goeldlin 2014** | Switzerland | Single arm study, pre- or post-test only | First-year medical students   - Pre-Evaluation: n=71 - Post-Evaluation: n=76 | - Clinical skills training - Problem-based courses | - *Attitudes, knowledge and clinical skills* | - Higher attitude score in students after training (p=0.062) - Significantly higher knowledge score after training (p<0.001) - 85.6% successfully passed OSCE skills examination |
| **Mappilakkandy 2014** | UK | Single arm study, pre-/post-test | Final-year medical students (n=29) | - CGA teaching blocks based on problem-based learning | - *Self-rated skills* | - Rating skills for doing a CGA improved from an average of 1.8/5 to 3.4/5. |
| **van de Pol 2014** | The Netherlands | Controlled, non-randomized study, pre-/post-test | Medical students   - Intervention: n=29 - Control: n=24 | - Elective game-based course in geriatrics   - Based on medical-teaching game GeriatriX - Control group: Students taking an elective neuroscience course | - *Self-perceived knowledge and attitudes* | - Significant post-intervention improvement in knowledge in 12 of 18 topics in intervention group, in 1 topic in control group (p<0.05) - Significant post-intervention change in attitudes in intervention group (p=0.02) compared to control group (p=0.30) |
| **Tam 2014** | Australia | Single arm study, pre-/post-test | Fifth-year medical students (n=54) | - Geriatric medicine course | - *Attitudes and self-perceived competency* | - Significant post-intervention improvement in attitude (p<0.001) and self-perceived competency (p<0.001) |
| **Atkinson 2013** | USA | Single arm study, pre-/post-test | Third-year medical students (n=112)   - Second post-evaluation at graduation: n=64 | - Geriatrics rotation | - *Knowledge and attitudes* | - Knowledge and attitudes improved significantly after the rotation - Significant post-intervention improvement of knowledge (p<0.001) which persisted at graduation. - Improvement in attitudes did not persist at graduation. |
| **Igenbergs 2013** | Germany | Controlled study, Cohort comparison (randomized) | Fourth-year medical students   - Intervention: n=106 - Control: n=100 | - Mandatory office-based general practice clerkship (one to one tutoring)   - Home visit to execute and interpret a CGA - Comparison of one group with supportive checklist with one group without checklist | - *Frequency of checklist item content addressed* - *Frequency of documented positive results in the medical care process* - *Comparison of achieved grades*   *(performance)* | - Significantly higher frequencies of documented positive results for 11 of 12 topics in intervention group (p<0.001) - Significantly better average of grades in intervention group (p<0.001) |
| **Strano-Paul 2011** | USA | Controlled, non-randomized study, post-test only | Third-year medical students (n=115) | - Ambulatory care clerkship - Variable clinical exposure:  Control groups = students with less or without additional clinical practice | - *Clinical skills, ability to perform geriatric assessments* | - No significant score differences between the groups - No significant association between OSCE scores and clinical practice/number of geriatric patients seen (p=0.255) |
| **Sutin 2011** | USA | Controlled, non-randomized study, pre-/post-test | Third-year medical students   - Intervention: n= 42 - Control: n=128 | - Standardized aging patient experience | - *Knowledge and clinical skills* | - Significant post-intervention improvement in knowledge in intervention group (p<0.02) - Intervention group scored significantly better in all three domains in geriatric OSCE station (p<0.001 – p<0.04) |
| **Diachun 2010** | UK & Canada | Controlled randomized trial (RCT), pre-/post test | Third-year medical students   - Intervention: n=149 - Control: n=113 - Pre- and post-evaluation (Intervention and control group): n=196 | - Intervention: Rotation with a combined geriatric medicine/geriatric psychiatry focus   - Small-group teaching sessions - Control: Rotation in otolaryngology/ ophtalmology | - *Knowledge, attitudes and clinical skills* | - Significant improvement of knowledge in intervention group compared to control group (p=0.004) - Worsening of attitudes towards older adults in both groups, but slightly less in intervention group - Significant higher scores (p<0.001) and higher pass rate (p<0.001) of intervention group in OSCE |
| **Zwahlen 2010** | USA | Cross-sectional | Medical students (n=347)   - Survey: at the beginning of medical school + at the end of the second year | - Curricular intervention with implementation of core geriatric content | - *Knowledge and attitudes* | - Significant post-intervention improvement of knowledge (p<0.001) - No significant post-intervention improvement of attitudes (p=0.21) |
| **Denton 2009** | USA | Controlled study, pre-/post test | Third-year medical students   - Intervention: n=17 - Control: n=16 | - Internet and CD-ROM-based geriatric self-study materials - Intervention: Geriatric home visit, reflective paper and discussion | - *Knowledge and attitudes* | - Geriatrics knowledge improved similarly in both groups (p=0.46) - Attitudes improved in the intervention group in three of four domains: geriatric patients in general (p=0.04), improved home care training (p=0.02), time and reimbursement for home visits (p=0.02) |
| **Fisher 2009** | USA | Single arm study, pre-/post-test | Third-year medical students (n=144) | - Intensive course in geriatric medicine | - *Attitudes/Learning experience* | - 97% of students agreed that learning how to care for older patients was important - 84% of students left course with better understanding of geriatric medicine - 74% of students felt more comfortable with addressing geriatric issues |
| **Oates 2009** | USA | Single arm study, pre-/post-test | Fourth-year medical students (n=58) | - Geriatric block rotation/clerkship | - *Perceived knowledge and abilities* | - Significant post-intervention improvement in perceived knowledge and abilities (p<0.001) |
| **Goldman 2008** | USA | Single arm study, pre-/post-test | Third-year family medicine students (n=86) | - Multiple format clerkship block: workshop, small-group case discussion, online video, online discussion | - *Competence and actual use of assessment tools* | - Significant post-intervention improvement of competence (p<0.05) - Most students performed the required assessment tools (MMSE 77%, GDS 84%, ADL 77%, Get-up-and-go 63%), with the exception of IADL (31%) |
| **Sanchez-Reilly 2007** | USA | Single arm study, pre-/post-test | Fourth-year medical students (n=25) | - Elective geriatric palliative medicine curriculum | - *Knowledge, attitudes and confidence in communication and behaviour* | - Significant post-intervention improvement in knowledge (p<0.001) - No significant changes in attitudes and confidence in communication as well as in self-reported behaviours |
| **Bates 2006** | USA | Single arm study, post-test only | First- and second-year medical students   - Focus group Evaluation: n=23 - Questionnaire Evaluation: n=48 | - Senior mentor program orientation | - *Knowledge and attitudes* | - Increased knowledge about geriatrics, specifically gait assessment, ADL evaluation, mental health screening and foot examination - Positive attitude change about geriatric patient care |
| **Roscoe 2005** | USA | Single arm study, pre- and/or post-test (time series design) | Third-year medical students   - Attitudes Evaluation: n=252 - Knowledge Evaluation: n=85 | - Geriatric medicine program | - *Knowledge and attitudes* | - Significant post-intervention improvement in attitudes, also 1 year post-intervention - Significant post-intervention improvement in knowledge (p<0.001) |
| **Struck 2005** | USA | Single arm study, pre-/post-test | Third-year medical students (n=135) | - Geriatric medicine clerkship | - *Knowledge, skills and attitude* | - Significant post-intervention improvement of knowledge (p<0.001) - 50% of students agreed or strongly agreed on improvement of physical examination skills and 66% agreed or strongly agreed on improvement of patient assessment skills - Attitude survey showed positive experience with intervention |
| **Newell 2004** | USA | Observational cohort study | First- and second-year medical students (n = n.a.) | - Infusion of geriatric content in a medical school curriculum | - *Knowledge and attitudes* | - Significant improvement in knowledge in 1^st^ year cohort compared to cohorts before enhanced geriatrics curriculum) - Significant difference on 7 of 20 knowledge items (p<0.05) - Significant difference on two attitudinal items (p<0.05) |
| **Duque 2003** | Canada | Controlled study (Multi-arm), post-test only | Second-year medical students (n=182) | - Module: Introduction to clinical medicine - Group 1: 10-half-day 10-week model - Group 2: 1 integrated week model | - *Knowledge* | - Significant difference between groups with 58% in group 2 giving a rating as very good or excellent and 51% group 1 giving a rating of fair or good (p<0.002) |
| **Flaherty 2002** | USA | Controlled study (Multi arm), pre-/ post-test | Third- and fourth-year medical students (n=325)   - School A: n=48 - School B: n=112 - School C: n=51 - School D: n=37 - School E: n=77 | - Home Care Rotation Program | - *Attitudes* | - Significant post-intervention improvement in attitude scores in three domains: general attitudes, home-based therapies and home care training - School programs’ characteristics significantly correlated with changes in total scores |
| ***Residency/Internship*** | | | | | | |
| **Thillainadesan 2023** | Australia | Single arm, pre- and/or post-test | Postgraduate (PGY 1-3) residents   - Pre-Evaluation: n=8 - Post-Evaluation: n=12 | - Microlearning and ward-based experiential methods | - *Performance: Occurrence of care processes* | - Occurrence of care processes significantly increased in 9 of 12 care processes (p=0.009 – p<0.001) |
| **Chao 2022** | USA | Single arm study, pre-/post-test | PGY-1 Internal medicine residents (n=22) | - 4 weeks core rotation alternating with 2 weeks of ambulatory care - Case-based, interactive teaching sessions | - *Confidence level in geriatrics content* - *Enhanced geriatric knowledge and skill levels* | - Significant post-intervention improvement of confidence (p<0.0001 – p=0.0007) - Significant post-intervention improvement of four knowledge and skill levels: functional assessment, geriatric pharmacotherapy, determination of eligibility for skilled/unskilled home services, and capacity determination (p<0.0001) |
| **Brown 2020** | USA | Single arm study, pre-/post-test | Family medicine and internal medicine residents (n=21) | - Interactive training program “Advanced Geriatric Evaluation Skills” | - *Knowledge* | - 90% of the residents improved the score from pre-test (average score 76%) to post-test (average score 88%) |
| **Ford 2019** | USA | Single arm study, post-test only | Family and internal medicine residents (n=353) | - Resident experience in geriatric medicine “Resident Award Summit” | - *Training impact* | - Event broadened knowledge of possible careers in geriatrics (M=4.66, SD=0.51) - Event would not necessarily result in pursuit of a fellowship in geriatrics (M=4.34, SD=0.80) - Event has positive impact on future practice (M=4.65, SD=0.58) |
| **Phillips 2019** | USA | Controlled, non-randomized study, pre-/post-test | Internal medicine residents   - Intervention: n=12 - Control: n=10 | - Workshop on core geriatrics topics for primary care   - Longitudinal patient case with unfolding information | - *Self-efficacy and knowledge* | - No statistically significant differences in self-efficacy or knowledge   - Increased post-intervention self-efficacy in intervention group |
| **Chang 2015** | USA | Single arm study, pre-/post-test | Second-year internal medicine residents (n=154) | - Ambulatory Geriatric Curriculum during block rotation | - *Knowledge, familiarity with geriatric assessment tools and self-efficacy/attitudes* | - Significant post-intervention improvement of knowledge in 7 of 9 questions (p<0.05 – p<0.001) - Post-test, more than 90% reported they were familiar with all screening assessments, know how to interpret results (p<0.002) and what to do with abnormal test (p<0.0001). More than 70% reported they were likely to use these geriatric assessments in their practice - Significant post-intervention improvement of comfort and self-efficacy (p<0.05 – p<0.001) |
| **Hogan 2014** | USA | Single arm study, pre-/post-test | Emergency medicine residents   - PGY-1: n=86 - PGY-2: n=88 - PGY-3: n=86 - PGY-4: n=41 | - Didactic intervention on Geriatric Emergency Medicine Competencies | - *Knowledge* | - Significant post-intervention improvement in all competency domains (p<0.001) |
| **Saffel-Shrier 2012** | USA | Single arm study, pre-/post-test | Second- and third-year family medicine residents (n=n.a.) | - Assisted living curricular variance with three half-day clinics each month | - *Knowledge, attitudes and competencies* | - No significant post-intervention improvement in knowledge and attitudes - Post-intervention improvement in all competencies, significant in 8 of 20 items (p<0.05) |
| **Ahmed 2011** | USA | Single arm study, pre-/post-test | Third-year internal medicine residents and fourth-year internal medicine and pediatric residents (n=26)   - Attitudes assessment: n=21 | - Geriatric orientation and rotation - Palliative orientation and rotation | - *Knowledge and attitudes* | - Significant post-intervention improvement of knowledge (p<0.001) - No significant post-intervention chance in attitudes (p=0.07-0.99) |
| **McLeod 2009** | USA | Controlled randomized trial (RCT) | First-year internal medicine residents   - Intervention PDA user with tool: n=20 - Control PDA non-user: n=34 - Control PDA user without tool: n=18 | - Geriatric assessment program primary care service rotation - Intervention: Geriatric assessment program with personal digital assistants - Control groups: Personal digital assistant non-user and personal digital assistant user without geriatric assessment tool | - *Knowledge* | - Significant post-intervention improvement of knowledge in all three groups (p<0.001; p<0.001; p=0.005) - No significant differences between groups |
| **Baum 2007** | USA | Single arm study, pre-/post-test | Internal medicine residents (n=67) | - Longitudinal long-term care rotation | - *Knowledge and attitudes* | - Significant post-intervention improvement of knowledge (p<0.001) and attitudes (p<0.001) |
| **Maurer 2006** | USA | Single arm study, pre-/post-test | Medical and psychiatry interns (n=46) | - Geriatric rotations | - *Knowledge, attitudes and assessment skills* | - Significant post-intervention improvement of attitudes (p=0.03), impressions (p<0.001), knowledge (p<0.001) and assessment skills (p<0.001) |
| **Steinweg 2001** | USA | Controlled, non-randomized study (time series design) | Second-year family practice residents   - Intervention: n=32 - Control: n=38 | - Geriatric block rotation | - *Knowledge* | - Significant post-intervention improvement of knowledge in intervention group (Geriatric Assessment Instrument, post-rotation and graduation scores p<0.001; Exam scores p=0.028) |
| ***Undergraduate and residency/internship*** | | | | | | |
| **McCrystle 2010** | USA | Single arm study, pre-/post-test | Internal medicine residents, Family practice residents, and medical students (n=23) | - Geriatrics rotation based on redesigned learner-centered geriatrics curriculum | - *Knowledge and competencies* | - Significant post-intervention improvement of knowledge (p<0.001) - Number of items rated with 0 (no exposure) or 1 (not competent) in self-competency checklist averaged 12.7 ± 7.9 in pre-test and 0.4 ± 1.0 in post-test |
| ***Postgraduate/Continuous professional development*** | | | | | | |
| **Eckstrom 2008** | USA | Single arm study, pre-/post-test | General internists (n=40) | - Continuing Medical Education Workshop | - *Knowledge and self-perceived competence* | - Significant post-intervention improvement in knowledge (p<0.0001) and self-perceived competence (11 of 14 items) |
| **Vass 2005** | Denmark | Controlled randomized trial (RCT) | Local general practitioners (n=n.a.) | - Education & short geriatric assessment program | - *Patient outcomes:*    - *Functional ability*   - *Mortality*   - *Nursing home admissions* | - Educational intervention was associated with improved functional ability (OR=1.20, 95%CI= 1.01-1.42, p=0.04), especially in 80-year olds (OR=1.53, 95%CI=1.12-2.09, p=0.008) - Intervention was not associated with mortality (RR=1.06, 95%CI=0.87-1.28, p=0.59) - Intervention was not associated with nursing home admissions (RR=0.74, 95%CI=0.50-1.09, p=0.13) |

**Table 2** Study characteristics, particular domains of CGA (n = 18 studies)

| **Author/Year** | **Country** | **Study Design** | **Study Population (and subgroups description)** | **CGA Domain and Education/Training Intervention** | **Primary outcomes of interest** | **Primary measures of effect** |
| --- | --- | --- | --- | --- | --- | --- |
| ***Undergraduate*** | | | | | | |
| **Hagiwara 2018** | USA | Single arm study, pre-/post-test | First-year medical students (n=27) | *Cognitive and functional assessment*   - Early-exposure geriatrics curriculum | - *Knowledge, attitudes and clinical skills* | - Significant post-intervention improvement of knowledge (p=0.01) and attitude (p<0.01) - Students met the standard of 85% of possible points in all OSCE cases |
| **Omlor 2016** | USA | Single arm study, pre-/post-test (retrospective) | Medical students (n=199) | *Gait and falls risk assessment*   - Intern Boot Camp: Session with three stations | - *Knowledge, ability and confidence in skills* | - Significant post-intervention improvement in all survey items comprising knowledge, ability and confidence in skills (p<0.0001 – p<0.001) |
| **Demons 2014** | USA | Single arm study, pre-/post-test (retrospective) | Third-year medical students (n=63) | *Falls risk assessment*   - Fall prevention program | - *Self-rated confidence in performing skills* | - Significant post-intervention improvement in all items comprising confidence in performing elements of a fall risk assessment (p=0.001 – p=0.004) |
| **Rughwani 2014** | USA | Controlled study (Multi arm), pre-/post-test | Third-year medical students   - Unfolding long case: n=43 - Case vignettes: n=38 | *Functional assessment*   - Two instructional methods in inpatient geriatrics: Long case vs. Case vignettes | - *Knowledge and knowledge retention* | - Significant improvement from pre-intervention to immediate post-intervention (p<0.001) and 1-year post-intervention (p<0.001) in both groups - No significant change from immediate post-intervention to 1-year post-intervention |
| **St. Onge 2013** | Canada | Cohort Comparison study, | Final-year medical students   - With prior mandatory geriatrics rotation: n=62 - Without prior mandatory geriatrics rotation: n=74 | *Functional and cognitive assessment*   - Mandatory geriatrics rotation | - *Performance and patient outcomes* | - Students with geriatrics exposure were more likely to assess function of older patients (27% vs. 12%, OR: 2.73, 95% CI: 1.12-6.66) - No other significant differences in performance - Patients of students with geriatrics exposure were less likely to die or be institutionalized (10% vs. 31%, OR: 0.24, 95% CI: 0.09-0.63) and had shorter lengths of stay (95% CI: -12.2- -2.07) |
| **Ouchida 2009** | USA | Single arm study, pre-/post-test | Third-year medical students (n=not stated) | *Functional assessment*   - Fast forward rounds (lecture, interactive video, small-group discussion, team-based learning exercise) | - *Knowledge, attitudes and behaviours* | - Significant post-intervention improvement of knowledge, attitudes and behaviours in all domains (transitional care, functional assessment, interdisciplinary team, community resources, reimbursement), with largest improvements in transitional care |
| **Duke 2009** | USA | Single arm study, pre-/post-test | First-year medical students (n=55) | *Functional assessment*   - Geriatric mentoring program | - *Attitudes* | - Statistically post-intervention improvement of in total survey scores (p=0.004) - Item-by-item analysis: Overall post-intervention improvement in 10 out of 11 items with significant improvement in 2 items (p<0.001; p=0.006) |
| **Tung 2008** | USA | Controlled, non-randomized study, post-test only | Third-year medical students   - Intervention: n=31 - Control: n=19 | *Functional assessment*   - Geriatric Home-based Assessment module - Control: Students without geriatric home-based assessment module experience | - *Knowledge, attitudes and clinical skills* | - Significant post-intervention improvement of knowledge (p<0.001) and performance of geriatric assessments in community (p<0.001) - Significantly higher OSCE station score in the intervention group (p<0.001) |
| **Adelman 2007** | USA | Single arm study, post-test only | First-year medical students (n=311) | *Functional assessment*   - Introduction to the geriatric patient module | - *Knowledge, skills, attitudes* | - Most students (99%) agreed that meeting the older patient in the small group was worthwhile, whereas 98% students believed that it helped contribute to their understanding about caring for older people. - Students are made aware of ageist behaviour (98%) and the importance of effective communication skills (99%). - Students agreed that they had learned basic components of enhanced social history (71% strongly agreed) and the Simple Geriatrics Screen (65.5% strongly agreed). However, they were less confident about conducting a social history (56%) or administering Simple Geriatrics Screen (38%) |
| **Supiano 2007** | USA | Observational cohort study | Third-year medical students   - Evaluation Knowledge: n=621 - Evaluation Skills: n=505 | *Functional assessment*   - Vertically integrated geriatric curriculum | - *Knowledge (longitudinal) and clinical skills* | - Significant post-intervention improvement in knowledge since curriculum changes (p<0.001) - Significant progressive post-intervention improvement in clinical skills (OSCE) since curriculum changes (p<0.001) |
| **Ruiz 2007** | USA | Single arm study, pre-/post-test | Second-year medical students   - Evaluation online training: n=95 - Competency assessment: n=137 | *Mobility assessment*   - Interactive e-learning tutorial | - *Attitudes, self-efficacy, knowledge and competency/skills* | - Significant post-intervention improvement of self-efficacy (p<0.001) - Moderate correlation of post-rating of self-efficacy and knowledge (p=0.023) - Mean competency assessment score was 16.9 out of 18 (SD=1.1) - Significant difference in verbal instructions of competency assessment of tutorial group vs. no-tutorial group (p=0.021), no significant difference in guarding or scoring domain |
| **Williams 2006** | USA | Single arm study, post-test only | First-year medical students (n=171) | *Functional assessment*   - Geriatrics Standardized Patient Instructor | - *Performance and learning experience* | - High post-intervention performance scores with mean scores of 92±10 for functional assessment and 93±7 for communication skills - Significant increases in comfort with performing functional assessment (p<0.01) |
| **Powers 2002** | USA | Single arm study, post-test only | Third-year medical students (n=135) | *Cognitive assessment*   - Mandatory geriatrics clerkship | - *Clinical skills, Performance, cognitive knowledge* | - Student performance in various clinical areas (hospice mean score 89%, outpatient geriatric clinic mean score 86%, transitional care mean score 79%, nursing home mean score 77%) was higher than student performance in the OSCE (mood disorder mean score 71.34%, cognitive dysfunction mean score 73.72%) - Students found the rotation gave the, valuable information on older patient care and the interdisciplinary approach; they believe that they would be able to incorporate their geriatrics learning into future practice. |
| **Penn 2001** | USA | Single arm study, pre-(post-test) | Third-year medical students (n=varied because of missing data) | *Functional assessment*   - Geriatric assessment experience | - *Knowledge, comfort and experience* | - Significant post-intervention increase in students’ levels of knowledge, comfort and experience with CGA, physical function, selection of CGA patients, coordination of health care team, functional problem list, management of frail elderly (p<0.0001) |
| **Swagerty 2000** | USA | Single arm study, pre-/post-test | Third-year medical students (n=130) | *Functional assessment*   - Case-oriented, web-based curriculum in geriatric medicine | - *Knowledge* | - Average post-intervention increase of knowledge scores of 13 items |
| ***Residency/Internship*** | | | | | | |
| **Moriarty 2018** | USA | Single arm study, pre-/post-test | Primary care residents   - Evaluation Attitudes: n=31 - Evaluation Confidence: n=28 | *Mobility and cognitive assessment*   - Connecticut Older Adult Collaboration for Health: Integration of a geriatrics curriculum into core internal medicine residency curriculum | - *Attitudes and self-rated confidence performing skills* | - Significant post-intervention change in attitude that medical care for the elderly uses up too many resources (p=0.03), other attitudes remained positive - Significant post-intervention improvement of confidence in managing patients with cognitive impairment (p=0.01), conducting mobility assessments (p<0.01), providing medication management (p<0.01), conducting goals of care discussions (p<0.01) and managing and treating patients with Alzheimer’s or related dementias (p=0.01) |
| **Caton 2011** | USA | Single arm study, pre-/post-test | Internal medicine residents (n=51) | *Falls assessment*   - Multicomponent program (lecture, academic detailing, case studies) | - *Knowledge and confidence* | - Significant post-intervention increase in number of residents with a knowledge score of 75% (p<0.001) - Significant post-intervention improvement in confidence to identify an older adult at risk for falls and perform a fall history (p=0.004) and to perform a focused examination (p<0.001) |
| **Robbins 2002** | USA | Single arm study, post-test only | Family medicine residents (n=3) | *Functional assessment*   - Expanded geriatric curriculum with three-part teaching series | - *Performance(skills)* - *Attitude (change in bias)* | - All residents were able to achieve an observer rating score greater than 70% for completion/ performance of a geriatric functional assessment - Although little changes in attitude could be observed, an impact of negative bias (attitudinal change) could not be clearly demonstrated. |
